# Editorial: Challenges in Computational Enzymology

**DOI:** 10.3389/fchem.2019.00690

**Published:** 2019-10-22

**Authors:** Vicente Moliner, Fahmi Himo

**Affiliations:** ^1^Departamento de Química Física y Analítica, Universidad Jaume I, Castellón de la Plana, Spain; ^2^Arrhenius Laboratory, Department of Organic Chemistry, Stockholm University, Stockholm, Sweden

**Keywords:** computational chemistry, enzyme catalysis, DFT, QM/MM, molecular modeling, molecular dynamics

Living organisms utilize enzymes to accelerate and control chemical reactions, and the attraction of these biological catalysts lies not only in their extraordinary efficiency, but also in their selectivity and their ability to function at mild conditions. Because of their involvement in fundamental biological processes, enzymes are common targets for the development of drug molecules in the pharmaceutical industry. In addition, enzymes are today increasingly employed as synthetic tools for industrial production of high value chemicals. It is therefore of great importance to understand in depth how these fascinating molecular machines perform their reactions, both from a fundamental scientific point of view and also for technical applications. To this end, computational approaches have in recent years become an indispensable tool in this endeavor. A number of powerful methodologies have been developed that have allowed for breakthroughs in the mechanistic understanding of enzymes. The exponential growth of computer power has, of course, been of key importance here. The field has also benefited greatly from the fruitful collaborations between theoreticians and experimentalists, where results derived from the simulations are used to interpret the experiments, and also to predict the behavior of natural and altered enzymes.

This Research Topic is concerned with the general theme of computational enzymology. It consists of 13 contributions covering various aspects of the field, spanning from benchmarking of methods to high-end applications and reviews of recent work.

In a review paper by Wei et al., the authors summarized their work on the modeling of metalloenzymes with both the quantum chemical cluster approach and the hybrid quantum mechanics/molecular mechanics (QM/MM) method. This review discussed recent progress in the computational understanding of mechanisms and various selectivities, such as chemo-, regio-, and stereoselectivity, in this important class of enzymes. In a more focused mini-review, Caddell Haatveit et al. described a computational protocol for modeling cytochrome P450 and exploring its reactivity and selectivity. The approach leads to predictions of enzyme variants that are more efficient and selective. Rovaletti et al. discussed in another mini-review the modeling of an illustrative example of redox-active enzymes, namely Mo/Cu-dependent CO dehydrogenase. The challenges concerned with both the DFT and the QM/MM approaches are highlighted and promising future directions are mentioned. In the paper of Siegbahn and Blomberg, problems associated with the study of mechanisms of redox processes in enzymes by DFT methods were discussed. An alternative systematic approach was proposed, where the amount of exact exchange in the B3LYP functional can be used as a parameter, rather than using a large number of different functionals. The limitations of DFT methods to study catalytic reaction mechanisms involving Fe(III)/Fe(II) oxidation states were discussed in the paper of Listyarini et al. The benchmark study, based on QM and QM/MM computational methods, provides guidelines to estimate the inaccuracies coming from the density functionals and to choose the most appropriate ones.

On the applications side, a number of very interesting papers are included in the Research Topic. Marques et al. presented a molecular dynamics study, employing metadynamics and adaptive sampling techniques, to estimate the rates for unbinding of the product molecule from the enzyme haloalkane dehalogenase DhaA and its mutant DhaA31. The simulations provided insights into the energetic bottlenecks in the rate-limiting unbinding process, which is of great help for the design of improved biocatalysts. Alonso-Gil et al. unraveled the hydrolytic reaction mechanism of *Neisseria polysaccharea* amylosucrase (NpAS), a member of GH13 family, based on QM(DFT)/MM metadynamics simulations. The results provide an atomistic picture of the active site reorganization along the catalytic double-displacement reaction, consistent with the general conformational itinerary observed for α-glucosidases. Kumar et al. investigated the binding modes of two inhibitors of the two metalloproteases (human JAMM deubiquitinylases Rpn11 and CSN5) using MD simulations and binding energy analysis and found that it was necessary to include larger heterodimeric protein-protein complexes in order to avoid unrealistic structural changes.

Alonso-Cotchico et al. combined force field-based techniques with a variety of trajectory convergence analyses to study the effect of cofactor binding on the conformational plasticity of the Lactococcal multidrug resistance regulator (LmrR) protein, a crucial aspect in order to design efficient artificial metalloenzymes. Martí et al. used QM/MM calculations to explore the mechanisms of the inactivation process of kanamycin A catalyzed by 4′-O-nucleotidyltransferase. Free energy perturbation techniques were employed and primary and secondary ^18^O kinetic isotope effects were computed and compared to available experiments to elucidate the detailed reaction mechanism. Romero-Téllez et al. used QM(DFT)/MM calculations to compare the glycosylation, hydrolysis and transglycosylation steps catalyzed by wild type *Thermus thermophilus* β-glycosidase. They showed how the molecular understanding of similarities and differences between hydrolysis and transglycosylation steps may be of help in the design of new biocatalysts for glycan synthesis. Prejanò et al. described based on QM and QM/MM calculations the reaction mechanism of the inhibition mechanism of hydrolyzed piperlongumine (hPL), an anticancer compound whose activity is related to the inhibition of human glutathione transferase of pi class (GSTP1). Finally, Timmins et al. studied the reaction mechanism of the HctB, a non-heme iron halogenase, using a combination of MM, MD, QM/MM, and DFT techniques. The effect of the substrate position in the active site on the halogenation vs. hydroxylation selectivity is discussed and comparison is made to other non-heme iron enzymes.

We believe that the new insights presented in these contributions will lead to advances in both fundamental understanding and practical applications in, for example, the design of new drug compounds and the developments of new biocatalysts for the chemical industry. We wish finally to thank all the contributors for their excellent work and all the reviewers for their comments that helped to improve the contents.

## Author Contributions

All authors listed have made a substantial, direct and intellectual contribution to the work, and approved it for publication.

### Conflict of Interest

The authors declare that the research was conducted in the absence of any commercial or financial relationships that could be construed as a potential conflict of interest.

